# Sodium carbonate and sodium silicate promote the Ca-montmorillonite: the nucleation, stabilization and hydrophilicity mechanisms

**DOI:** 10.3389/fchem.2025.1646971

**Published:** 2025-09-04

**Authors:** Chenglong Yin, Jin Huang, Shao-yu Deng, Chong Ma, Yong Peng

**Affiliations:** 1 School of Urban Construction, Zhejiang Shuren University, Hangzhou, China; 2 College of Civil Engineering and Architecture, Zhejiang University, Hangzhou, China

**Keywords:** stabilization and hydrophilicity mechanism, Ca-montmorillonite, density functional theory (DFT), Na_2_CO_3_ and Na_2_SiO_3_, soil stabilization, calcium-based stabilizer

## Abstract

Montmorillonite is widely utilized in catalysis, environmental science, and civil engineering. Previous studies have demonstrated that Na_2_CO_3_ and Na_2_SiO_3_ enhance the stability of Ca-montmorillonite-rich clayey soils in chemical soil stabilization. However, the microscopic mechanisms underlying their effects on nucleation, stabilization, and hydrophilicity remain unclear. This study investigates these mechanisms using Scanning Electron Microscopy (SEM) and Density Functional Theory (DFT) calculations. SEM results show that Na_2_CO_3_ and Na_2_SiO_3_ enhance the strength of the stabilized soils by promoting the formation of cementitious and crystalline substances. DFT calculations reveal that SiO_3_^2-^ and CO_3_^2-^ exhibit the most negative adsorption energies of −6.2 eV and −5.1 eV, respectively, in the exchangeable layers of montmorillonite, significantly higher than those of Na^+^ and Ca^2+^. On the montmorillonite surface, SiO_3_^2-^ and CO_3_^2-^ display even lower adsorption energies of −8.7 eV and −6.8 eV, respectively. Water molecules preferentially adsorb dissociatively on the montmorillonite surface with an energy of −3.1 eV; however, their adsorption is suppressed following the adsorption of Ca^2+^, Na^+^, CO_3_^2-^, and SiO_3_^2-^, with energies decreasing to between −1.1 eV and −2.5 eV. Differential charge density plots indicate that ion adsorption leads to charge redistribution and the formation of chemical bonds. Specifically, Ca^2+^ and Na^+^ donate cationic charge, while CO_3_^2-^ and SiO_3_^2-^ accept electrons. The study further explains why Na_2_CO_3_ and Na_2_SiO_3_, in combination with lime, are more effective than lime alone in soil stabilization. A mechanism model for nucleation, stabilization, and hydrophilicity is proposed to explain the role of Na_2_CO_3_ and Na_2_SiO_3_ in promoting Ca-montmorillonite stabilization. This work provides valuable insights into the chemical properties of montmorillonite and the synergistic effects of calcium-based stabilizers combined with Na_2_CO_3_ and Na_2_SiO_3_ for soil stabilization.

## 1 Introduction

Clayey soil is a naturally occurring, multi-scale, and multi-phase mixture formed through geological and biological cycles. Its primary inorganic components are clay minerals—such as montmorillonite, illite, and kaolinite—which are derived from the weathering of rocks during the geological cycle and typically appear as fine, plate-like sheets (Giese and Van Oss, 2002; Mitchell and Soga, 2005). On one hand, these clay minerals possess a large specific surface area and interlayer ion exchange capacity, which make them widely applicable as adsorbents (Zhu et al., 2016), catalysts (Huang et al., 2023), and coagulants (França et al., 2022) in the field of environmental pollution control and remediation. On the other hand, the surfaces of these minerals are generally negatively charged and exhibit high surface free energy, resulting in a strong affinity for water and causing varying degrees of volume expansion upon moisture exposure (Mitchell and Soga, 2005; Petry and Little, 2002). This pronounced sensitivity to moisture fluctuations of clay minerals leads to significant changes in the volume and mechanical strength of clayey soils, thereby posing risks to the stability and safety of buildings and infrastructure constructed on them. Such instability not only compromises structural integrity and public safety but also leads to substantial annual maintenance costs, limiting the broader use of clayey soils—especially those with relatively high water content—in civil engineering applications (Anburuvel, 2023; Pup and pala, 2016).

Soil stabilization is a widely adopted and cost-effective technique for enhancing the engineering properties of fine-grained soils, particularly clayey soils (Anburuvel, 2023; Barman and Dash, 2022; Pup and pala, 2016). This approach typically involves blending various stabilizers into the soils, followed by compaction at an optimal moisture content and a subsequent curing period (Yin et al., 2018; Zada et al., 2023). The fundamental mechanism of soil stabilization relies on utilizing the chemical and/or physicochemical reactions occurred in the stabilizer-soil mixture to modify the surface characteristics and interfacial interactions of soil particles, thus improving the strength, water-resistance and other engineering properties of the soils (Anburuvel, 2023; Barman and Dash, 2022; Mitchell and Soga, 2005; Pup and pala, 2016).

A wide variety of materials have been employed for soil stabilization. Among them, lime and Portland cement—both containing calcium and thus referred to as calcium-based stabilizers—have been among the earliest and most extensively used, due to their widespread availability and well-documented effectiveness (Bell, 1996; Ma et al., 2018; Raja and Thyagaraj, 2020). The stabilization mechanisms of these two calcium-based stabilizers involve a series of reaction processes, including cation exchange, flocculation and agglomeration (particle restructuring), cementitious hydration, hardening, carbonation, and pozzolanic reactions (Anburuvel, 2023; Barman and Dash, 2022; Jiang et al., 2021; Pup and pala, 2016; Wang et al., 2023; Yu et al., 2024).

The introduction of different additives into the calcium-based stabilizer-soil system can alter the reaction processes, resulting in varying stabilization effects. It has been reported that sodium carbonate (Na_2_CO_3_) and sodium silicate (Na_2_SiO_3_) can significantly enhance the strength, water resistance, and other properties of calcium-based stabilized soils (Dengliang and Aimin, 1988; Murmu et al., 2020; Rivera et al., 2020; Yin et al., 2025). Zhang et al. (Dengliang and Aimin, 1988) conducted a systematic evaluation of the effects of lime-fly ash-based stabilizers with various additives on the compressive strength of stabilizer-soil mixtures. Their results showed that sodium carbonate, sodium silicate, sodium hydroxide, sodium sulfate, and sodium phosphate notably improved the compressive strength of lime-fly ash soils. In our previous study (Yin et al., 2025), we also found that adding only 0.05% sodium carbonate and 0.05% sodium silicate, based on the dry soil weight in solution form, effectively enhanced the 7-day unconfined compressive strength of a calcium-based stabilizer-soil mixture, with the highest strength improvement observed at 34.5%.

The mechanism behind this phenomenon is not yet fully understood. Some researchers have attributed it to the reactions between ${\text{Ca}\left(\text{OH}\right)}_{2}$ and Na_2_CO_3_ or Na_2_SiO_3_, as described by the following equations:

$${\text{Ca}\left(\text{OH}\right)}_{2}+{\text{Na}}_{2}{\text{CO}}_{3}\;\to {\text{CaCO}}_{3}\;↓+2\text{NaOH}$$ (1)

$${\text{Ca}\left(\text{OH}\right)}_{2}+{\text{Na}}_{2}{\text{Si}O}_{3}\;\to {\text{CaSi}O}_{3}\;↓+2\text{NaOH}$$ (2)

The ${\text{Ca}\left(\text{OH}\right)}_{2}$ is derived from lime and the hydration of Portland cement. The resulting precipitates, calcium carbonate (CaCO_3_) and calcium silicate hydrate (C-S-H), enhance soil stabilization by modifying particle surface characteristics (Chung et al., 2021; Teerawattanasuk and Voottipruex, 2019), filling interparticle voids (Phummiphan et al., 2016; Teerawattanasuk and Voottipruex, 2019), and strengthening interparticle bonding (Abdullah et al., 2021). These processes collectively contribute to the improved macroscopic strength and water resistance of calcium-based stabilized soils. However, the atomic-scale mechanisms that underlie the strength enhancement and hydrophilicity regulation remain poorly understood, particularly in terms of the roles of Na_2_CO_3_ and Na_2_SiO_3_ in promoting Ca^2+^-clay coordination, modifying the adsorption sites of water molecule, and altering surface charge distribution.

The first-principles method, particularly Density Functional Theory (DFT), is a powerful computational approach for investigating the interaction mechanisms between materials at the atomic and electronic levels. Unlike molecular dynamics (MD) or Monte Carlo (MC) simulations, which primarily rely on classical force fields to model interactions, DFT provides a more fundamental understanding by directly calculating the electronic structure of a system. This allows for deeper insights into the nature of chemical bonds, reaction mechanisms, and material properties. As a result, DFT is particularly well-suited for exploring the interaction mechanisms between clay minerals and various materials. Montmorillonite, with its characteristic 2:1 layered structure and notable properties such as strong water absorption and swelling behavior, is often the preferred clay mineral for DFT-based calculations. Peng et al. (2016) demonstrated that water molecules exhibit distinct adsorption behaviors on different montmorillonite surfaces. On the Na-montmorillonite (001) basal surface, adsorption primarily occurs through electrostatic interactions between water molecules and Na^+^ cations. In contrast, on the (010) edge surface, hydrogen bonds form between water molecules and surface -OH or -OH_2_ groups. Miyamoto et al. (Chatterjee et al., 1999) further showed that Na^+^ cations migrate toward the negative charge centers of clay clusters, with each Na^+^ cation being coordinated by five interlayer water molecules in montmorillonite. Additionally, studies have explored the adsorption of water molecules by various interlayer cations in montmorillonite (Li et al., 2023), the adsorption of bisphenol by montmorillonite (Guo et al., 2022), and the acid activation process of montmorillonite (Fonseca et al., 2018). While previous studies have focused on water adsorption in montmorillonite, the use of DFT calculations to investigate the molecular nucleation mechanisms of stabilizer-montmorillonite mixtures and their subsequent effects on water adsorption remains limited. Understanding the nucleation and hydrophilicity mechanisms of these mixtures is crucial for gaining a deeper insight into the soil stabilization process. Therefore, this study employs the DFT method to explore these mechanisms.

Based on our preliminary experimental research (Yin et al., 2025), the aim of this study is to investigate the mechanisms by which Na_2_CO_3_ and Na_2_SiO_3_ enhance the stability of calcium-based stabilizer-soil mixtures. The morphological changes in the microstructure of the stabilized soils were investigated by SEM scanning. The interactions between Ca^2+^, Na^+^, CO_3_^2-^ and SiO_3_^2-^ with montmorillonite were examined using DFT calculations. In addition, the surface hydrophilicity before and after stabilization was analyzed, and the adsorption mechanism was explored through differential charge analysis. This work offers a theoretical perspective on the synergistic mechanisms of calcium-based stabilizers in combination with Na_2_CO_3_ and Na_2_SiO_3_ in soil stabilization.

## 2 Materials and computation details

### 2.1 Experimental materials

The experimental soil, a type of lean clay predominantly consisting of montmorillonite and other minerals, has its physical and chemical properties detailed in Table 1. For this study, the calcium-based stabilizer, denoted as B2, is a composite mixture of Portland cement (PC), lime (L), and fly ash (FA) with a mass ratio of 4:2:1(PC:L:FA), and the chemical compositions of these three components are illustrated in Table 2. The sodium carbonate ( ${{\text{Na}}_{2}\text{CO}}_{3}$ ) and sodium silicate ( ${{\text{Na}}_{2}\text{SiO}}_{3}\cdot {9H}_{2}O$ ) used in the experiments are all analytical pure chemical reagents produced by Shanghai Hushi Laboratory Equipment Co., Ltd (Shanghai, China).

**TABLE 1:** Physical and chemical properties of soil sample.

Natural dry density (g/cm^3^)	Dried moisture content (%)	Specific gravity	Liquid limit (%)	Plastic limit (%)	Plasticity index	Activity of clay	pH (distilled water)	pH (1M KCl solution)	Carbon content Wt (%)	Sulphur content Wt (%)
1.64	2.94	2.69	37.8	19.3	18.5	2.02	6.55	5.86	1.59∼4.25	0∼0.77

**TABLE 2:** Chemical composition of the calcium-based stabilizers.

Materials	Chemical compositions (Mass fraction, %)								
	SiO_2_	Al_2_O_3_	Fe_2_O_3_	CaO	Na_2_O	K_2_O	MgO	TiO_2_	SO_3_
PC	18.04	8.79	4.96	54.14	0.12	0.32	3.56	-	1.77
L	-	-	-	86.26	-	-	0.68	-	-
FA	11.61	21.73	1.75	40.28	0.95	1.36	0.49	1.66	0.61

### 2.2 Experimental methods

The experimental procedures primarily consisted of compaction testing, specimen preparation and curing, and unconfined compression testing, conducted in accordance with the Chinese Standard JTG 3441-2024. Initially, the B2 stabilizer and dried soil were thoroughly mixed in a mass ratio of 4:2:1:100 (PC:L:FA:dry soil), yielding a base mixture. Then the maximum dry density and optimum moisture content of the base mixture were tested to be 1.79 g/cm^3^ and 15%, respectively, by the modified Proctor compaction test. Subsequently, 0.05% sodium carbonate and 0.05% sodium silicate (calculated relative to the dry soil weight and added in an aqueous solution manner) were then incorporated into part of the base mixture to form a testing mixture. The mixtures with and without the two sodium salts were then statically compacted into cylindrical specimens, each measuring 50 mm in height and 50 mm in diameter, at their optimum moisture content using a 30 kN hydraulic pressing machine. Then, the compacted specimens were transferred to a curing room maintained at a temperature of 20 °C ± 1 °C and a relative humidity of 95% ± 5%. After a 7-day curing period, unconfined compression tests were conducted using a 30 kN hydraulic pressing machine at a displacement rate of 1 mm/min.

### 2.3 Experimental characterization

After the compression tests, samples extracted from the tested specimens were subjected to SEM scanning. An FEI Quanta 650 FEG Environmental SEM (ESEM) was employed for the scanning process, operating within an acceleration voltage range of 200V to 30 kV and with a maximum beam current of 200 nA.

### 2.4 DFT computational method

CP2K (Kühne et al., 2020) was employed to carry out the theoretical calculations in the framework of density functional theory (DFT). CP2K employed two representations of the electron density: localized Gaussian and plane wave basis sets. For the Gaussian-based (localized) expansion of the Kohn–Sham orbitals, we used a library of contracted molecularly optimized valence double-zeta plus polarization basis sets (VandeVondele and Hutter, 2007), and the complementary plane wave basis set had a cutoff of 400 Rydberg for the electron density. The generalized gradient corrected approximation of Perdew, Burke and Ernzerhof (PBE) (Blöchl, 1994) was adopted to relax the geometric structures. The energy and force convergence criteria of the self-consistent iteration were set to 10–5 eV and 0.03 eV Å-1. The ions are calculated in a 15 Å × 15 Å × 15 Å cell. Since montmorillonite has layered structure, no additional vacuum layer was set, which is consistent with previously reported montmorillonite calculations (Li et al., 2023; Peng et al., 2016). During calculation, all atoms remain relaxed.

The adsorption energies were used to assess the adsorption capacity of the montmorillonite models for molecules and ions. The adsorption energy is defined by Formula 3, where ΔE represents the adsorption energy, while E (surf), E (ion), and E (ion/surf) denote the total energies of the surface, the free ion, and the surface with the ion, respectively. The more negative the adsorption energy in value, the greater the exothermic heat released, and the more stable the system becomes.

$$\Delta E=E\left(\text{ion}/\text{surf}\right)\;–\;E\left(\text{surf}\right)\;–\;E\left(\text{ion}\right)$$ (3)

### 2.5 Montmorillonite surface model

**FIGURE 1:**
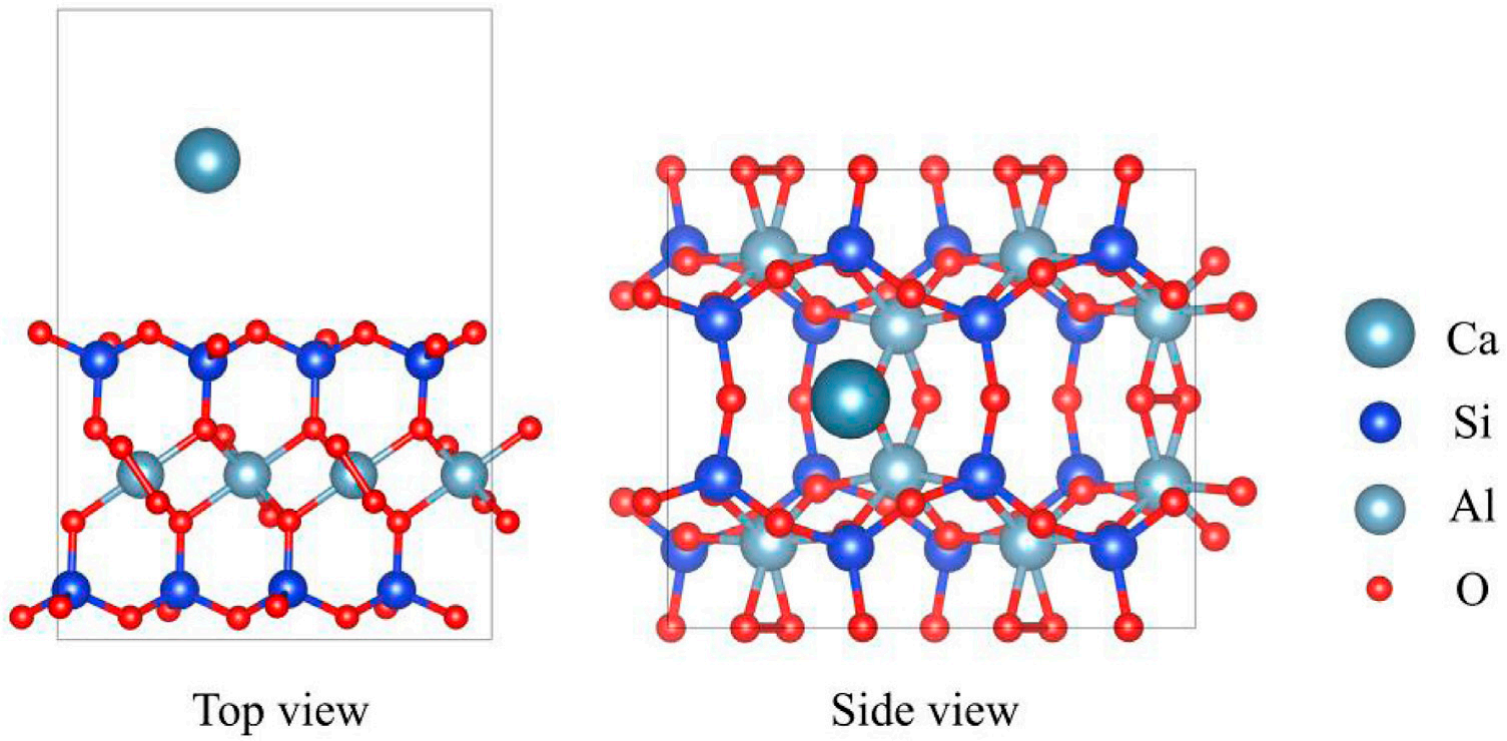
The top and side view of montmorillonite. The red, blue, gray, and dark gray represent O, Si, Al and Ca, respectively.

Montmorillonite (PDF#13-0135), with the chemical formula CaAl_4_Si_8_O_24_, belongs to the hexagonal crystal system. Figure 1 displays the top and side views of the montmorillonite model. The unit cell parameters are as follows: a = 5.169 Å, b = 5.169 Å, c = 15.02 Å, with angles α = 90.0°, β = 90.0°, and γ = 90.0°. In its structure, Ca^2+^ cations act as exchangeable ions located between the layers, while Al^3+^ cations reside in the bulk phase. The Si^4+^ cations are tetrahedrally coordinated within the SiO_4_ units, and the O^2-^ anions are 2-coordinated in the OSi_2_ environment and 3-coordinated in the OSiAl_2_ environment.

## 3 Results and discussion

### 3.1 The strength and microscopic morphological characteristics of the mixtures

The 7-day unconfined compressive strength of the base mixture was determined to be 1.182 MPa. After introducing 0.05% sodium carbonate and 0.05% sodium silicate, the 7-day unconfined compressive strength of the testing mixture escalated to 1.620 MPa, signifying a 34.5% enhancement in strength (Yin et al., 2025).

**FIGURE 2:**
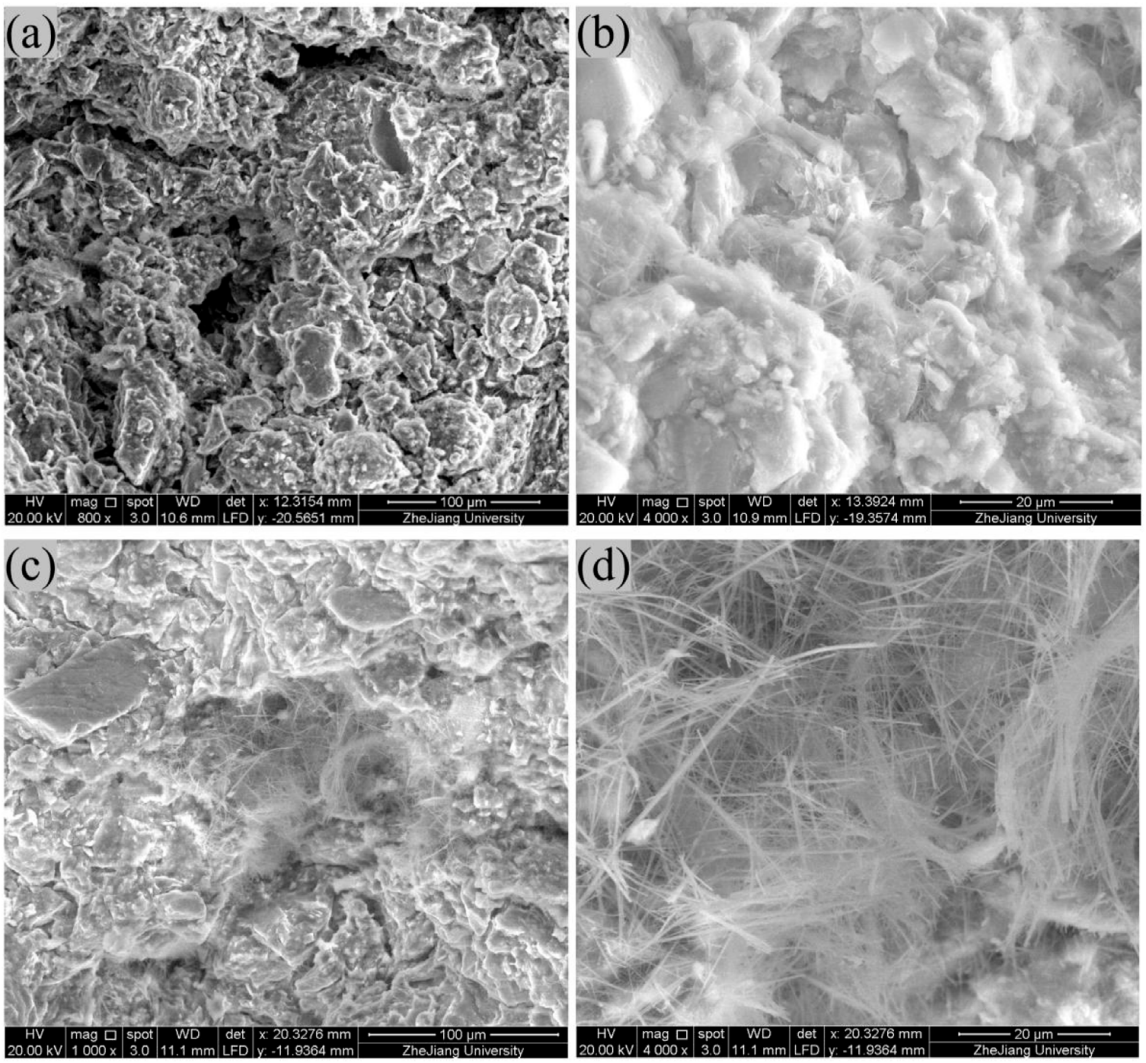
SEM images of soil samples **(a,b)** are the B2 stabilizer-soil mixture without Na_2_CO_3_ and Na_2_SiO_3_; **(c,d)** are the B2 stabilizer-soil mixture with the addition of Na_2_CO_3_ and Na_2_SiO_3_.

Figure 2 shows the SEM images of soil samples before and after the addition of Na_2_CO_3_ and Na_2_SiO_3_. In the SEM images of the B2 stabilizer-soil mixture, prominent pores can be observed, along with only a small amount of fibrous or reticular cementitious substances and acicular crystals on the particle surfaces (Cao et al., 2022; Wu et al., 2018). After adding Na_2_CO_3_ and Na_2_SiO_3_, the pores are filled with fibrous or reticular cementitious substances and acicular crystals, and similar deposits are observed coating the particle surfaces. This suggests that the addition of Na_2_CO_3_ and Na_2_SiO_3_ promotes the formation of cementitious and crystalline substances which are deduced to be C-S-H and calcium carbonate according to chemical reaction Equations 1, 2. These newly formed substances effectively modify the surface characteristics of the soil particles, fill interparticle voids, and enhance interparticle bonding. As a result, the strength of the stabilizer-soil mixtures increase from 1.182 MPa to 1.620 MPa, which aligns well with findings reported in previous studies (Abdullah et al., 2021; Chung et al., 2021; Dengliang and Aimin, 1988; Murmu et al., 2020; Phummiphan et al., 2016; Rivera et al., 2020; Teerawattanasuk and Voottipruex, 2019; Yin et al., 2025).

However, the existing techniques are difficult to characterize the microscopic processes and mechanisms of nucleation and crystallization of Na_2_CO_3_ and Na_2_SiO_3_ in the pores and on the surface of montmorillonite. Therefore, subsequently, the DFT method was adopted to study their nucleation and crystallization mechanisms as well as physicochemical properties.

### 3.2 Structures and adsorption energies of Ca^2+^, Na^+^, CO_3_^2-^ and SiO_3_^2-^

Montmorillonite contains both exchangeable layers and surfaces. Accordingly, the adsorption behaviors of Ca^2+^, Na^+^, CO_3_^2-^, and SiO_3_^2-^ in these two distinct scenarios were investigated.

#### 3.2.1 On exchangeable layer

**FIGURE 3:**
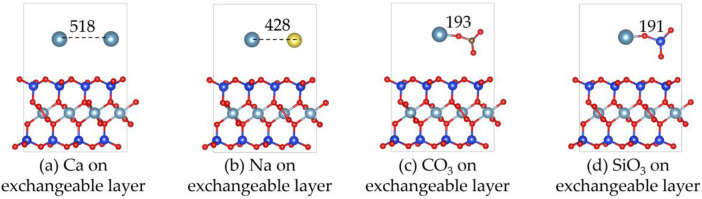
The structures of **(a)** Ca^2+^, **(b)** Na^+^, **(c)** CO_3_^2-^ and **(d)** SiO_3_^2-^ on the exchangeable layer of montmorillonite.

**FIGURE 4:**
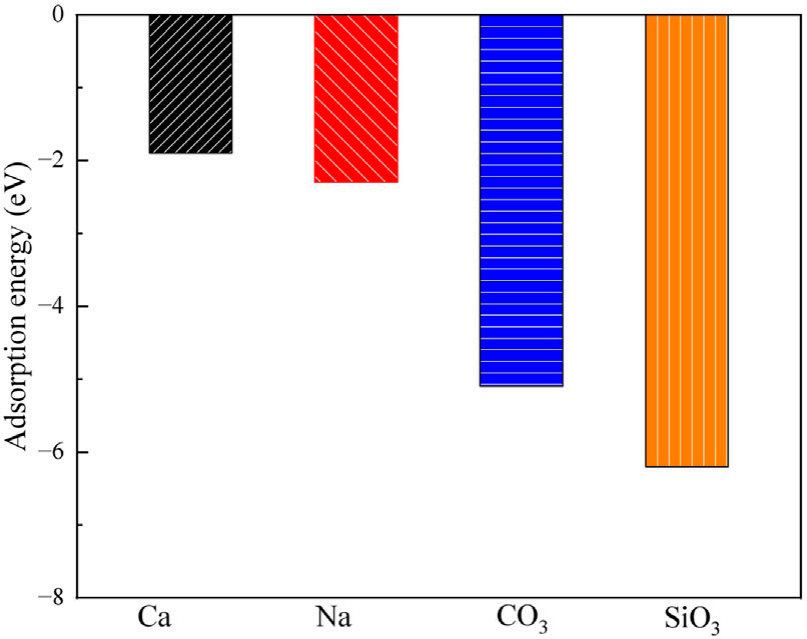
The adsorption energies of Ca^2+^, Na^+^, CO_3_^2-^ and SiO_3_^2-^ on the exchangeable layer of montmorillonite.

Figures 3, 4 show the structures and adsorption energies for the adsorption of Ca^2+^, Na^+^, CO_3_^2-^, and SiO_3_^2-^ on the exchangeable layer of montmorillonite. For Ca^2+^ adsorption on the exchangeable layer, the adsorption energy is −1.9 eV. The distance between the two Ca^2+^ atom is 518 pm. When Na^+^ adsorbs on the exchangeable layer, its adsorption energy is −2.3 eV, which is slightly higher than that of Ca^2+^ on exchangeable layer. The distance between the Ca^2+^ and Na^+^ is 428 pm.

For CO_3_^2-^ adsorption on the exchangeable layer, CO_3_^2-^ coordinates with Ca^2+^ through O. The Ca-O bond length is 193 pm, and the adsorption energy is −5.1 eV. For SiO_3_^2-^ adsorption on the exchangeable layer, SiO_3_^2-^ coordinates with Ca^2+^ through one O atom. The Ca-O bond length is 191 pm, and the adsorption energy is −6.2 eV.

Thus, SiO_3_^2-^ has the highest adsorption energies, indicating a stronger interaction with the montmorillonite exchangeable layer compared to CO_3_^2-^, Ca^2+^ and Na^+^. The adsorption energy order is SiO_3_^2-^ > CO_3_^2-^ >> Na^+^ > Ca^2+^. This implies that CO_3_^2-^ and SiO_3_^2-^ are more likely to be stably adsorbed on the layer than that of Ca^2+^ and Na^+^.

#### 3.2.2 On surface

**FIGURE 5:**
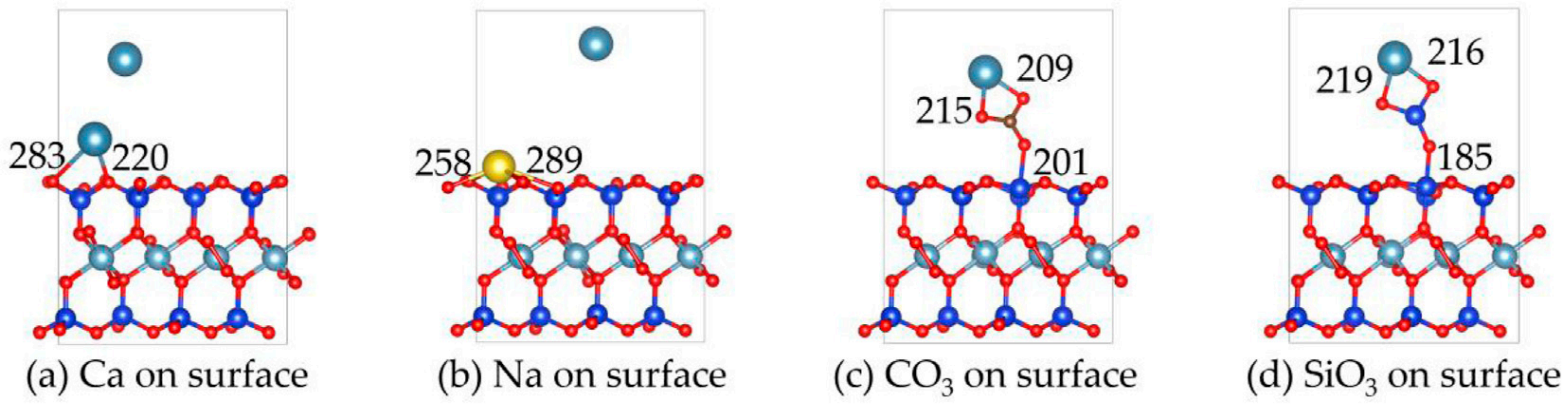
The structures of **(a)** Ca^2+^, **(b)** Na^+^, **(c)** CO_3_^2-^ and **(d)** SiO_3_^2-^ on the surface of montmorillonite.

**FIGURE 6:**
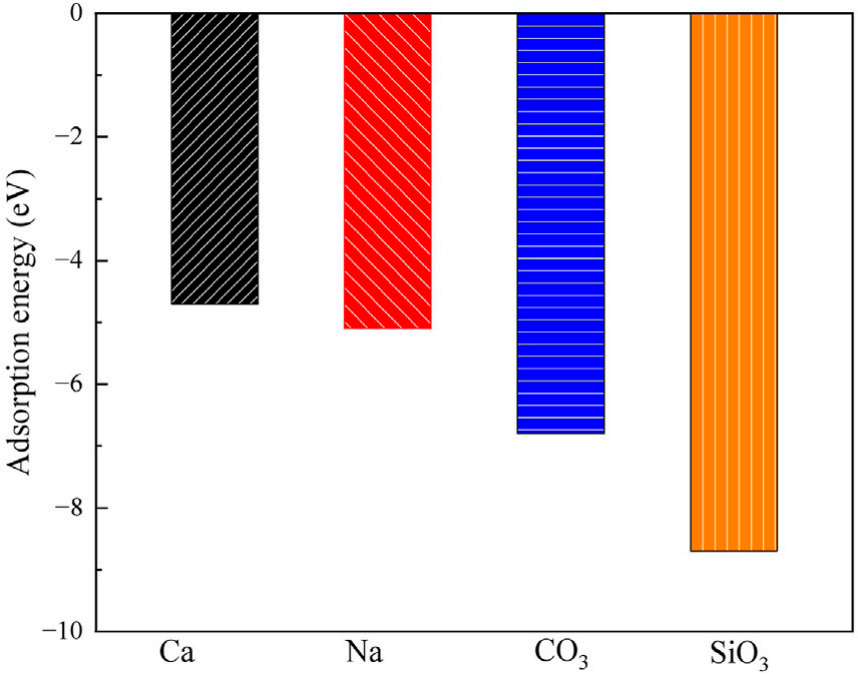
The adsorption energies of Ca^2+^, Na^+^, CO_3_^2-^ and SiO_3_^2-^ on the surface of montmorillonite.

Figures 5, 6 show the structures and adsorption energies for the adsorption of Ca^2+^, Na^+^, CO_3_^2-^, and SiO_3_^2-^ on the surface of montmorillonite. For the adsorption of Ca^2+^ on the surface of montmorillonite, the adsorption energy is - 4.7 eV Ca^2+^ adsorbs on the surface of montmorillonite by bonding with two O atoms. The bond lengths are 283 pm and 220 pm respectively. This indicates that for Ca^2+^, surface adsorption results in a higher adsorption energy and greater stability compared to layer adsorption. In contrast, during layer adsorption, its adsorption energy was only −1.9 eV. The higher adsorption energy during surface adsorption implies a stronger interaction between Ca^2+^ and the montmorillonite surface, thus making the adsorbed state more stable.

When Na^+^ adsorbs on the surface, its adsorption energy is −5.1 eV. The distances between two surface O atoms are 258 pm and 289 pm. The higher adsorption energy compared to Ca^2+^ suggests that Na^+^ has a stronger interaction with the surface.

For the adsorption of CO_3_^2-^ on the surface, the adsorption energy is −6.8 eV CO_3_^2-^ forms bonds with Ca^2+^ through two O atoms, with Ca-O bond lengths of 215 pm and 209 pm. Additionally, CO_3_^2-^ bonds with a surface Si atom via one O atom, with the O-Si bond length of 201 pm. For the adsorption of SiO_3_^2-^ on the surface, the adsorption energy reaches −8.7 eV, the highest among the four substances. SiO_3_^2-^ forms bonds with Ca^2+^ through two O atoms, with Ca-O bond lengths of 219 pm and 216 pm, and also bonds with a surface Si atom through one O atom, with O-Si bond length of 185 pm.

Overall, the order of adsorption energies is SiO_3_^2-^ > CO_3_^2-^ > Na^+^ > Ca^2+^. This implies that SiO_3_^2-^ is most likely to be stably adsorbed on the surface, followed by Na^+^ and CO_3_^2-^, while Ca^2+^ has relatively weaker adsorption stability.

The above study investigated the adsorption behavior of Ca^2+^, Na^+^, CO_3_^2-^, and SiO_3_^2-^. Both SiO_3_^2-^ and CO_3_^2-^ exhibite the most negative adsorption energies on the exchange layer and surface. This proves that Na^+^, CO_3_^2-^ and SiO_3_^2-^ all contribute to the solidification of Ca^2+^ and the stability of montmorillonite.

### 3.3 Hydrophilicity of montmorillonite

#### 3.3.1 Clean montmorillonite

**FIGURE 7:**
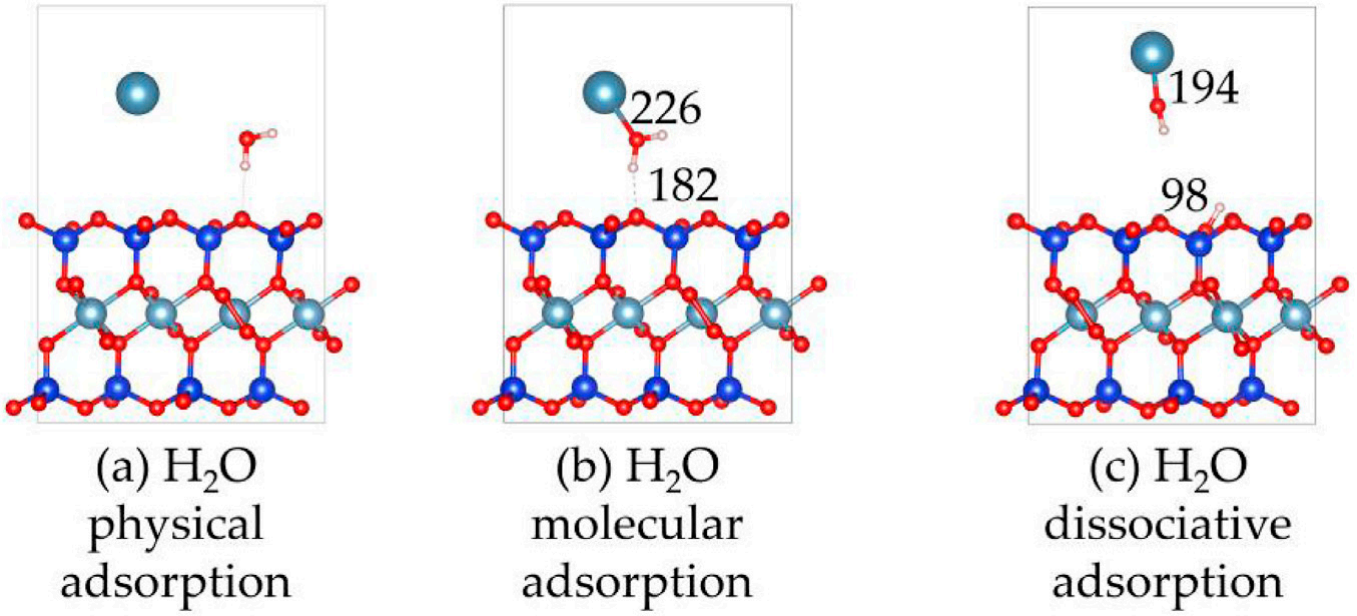
The structures of H_2_O and OH on the montmorillonite; **(a)** H_2_O physical adsorption, **(b)** H_2_O molecular adsorption, **(c)** H_2_O dissociative adsorption.

**FIGURE 8:**
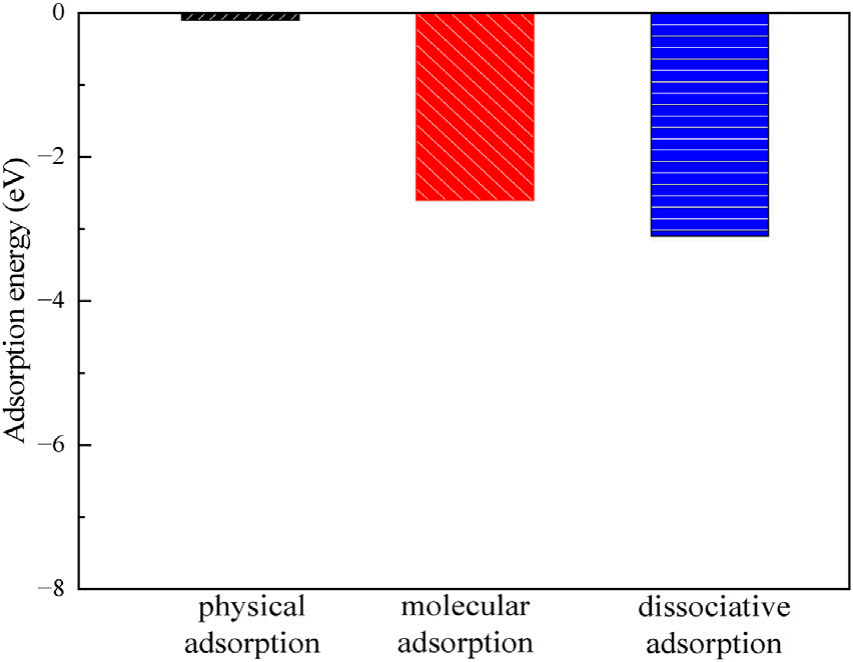
The adsorption energies of H_2_O and OH on the montmorillonite.

Figures 7, 8 show the structures and adsorption energies for the adsorption of H_2_O and OH on the montmorillonite. For the H_2_O physical adsorption configuration, the adsorption energy is only −0.1 eV. The H_2_O is through a hydrogen bond with the montmorillonite surface O, with the hydrogen bond distance of 195 pm. For the H_2_O molecular adsorption configuration, the adsorption energy is −2.6 eV. The H_2_O molecule bonds with the layer Ca^2+^, with the distance between the O atom of H_2_O and the layer Ca^2+^ atom being 226 pm. Additionally, H_2_O forms a hydrogen bond with the surface O atom, and the bond length of this hydrogen bond is 182 pm. This indicates that H_2_O can form a strong hydrogen bond with the montmorillonite surface. This strong hydrogen bond can further promote the dissociation of H_2_O. As shown in the H_2_O dissociative adsorption structure in Figure 7, the adsorption energy is −3.1 eV. The dissociated OH group bonds with layer Ca^2+^, with the Ca-O distance being 194 pm. The dissociated H atom bonds with a surface O atom, and the O-H bond length is 98 pm.

As can be seen from the above, H_2_O molecules are more likely to exist in a dissociated form on the surface of montmorillonite compared to molecular adsorption. Whether it is the dissociated adsorption of intact H_2_O molecules or the adsorption of a single OH group, they all adsorb onto the layer Ca^2+^ and tend to form hydrogen bonds with the surface. The formation of hydrogen bonds in all these cases further stabilizes the adsorbed species. Understanding these surface-water interaction mechanisms is of great importance for comprehending related chemical processes, such as ion exchange reactions occurring on the montmorillonite surface.

#### 3.3.2 Ca^2+^, Na^+^, CO_3_^2-^ and SiO_3_^2-^ absorbed montmorillonite

**FIGURE 9:**
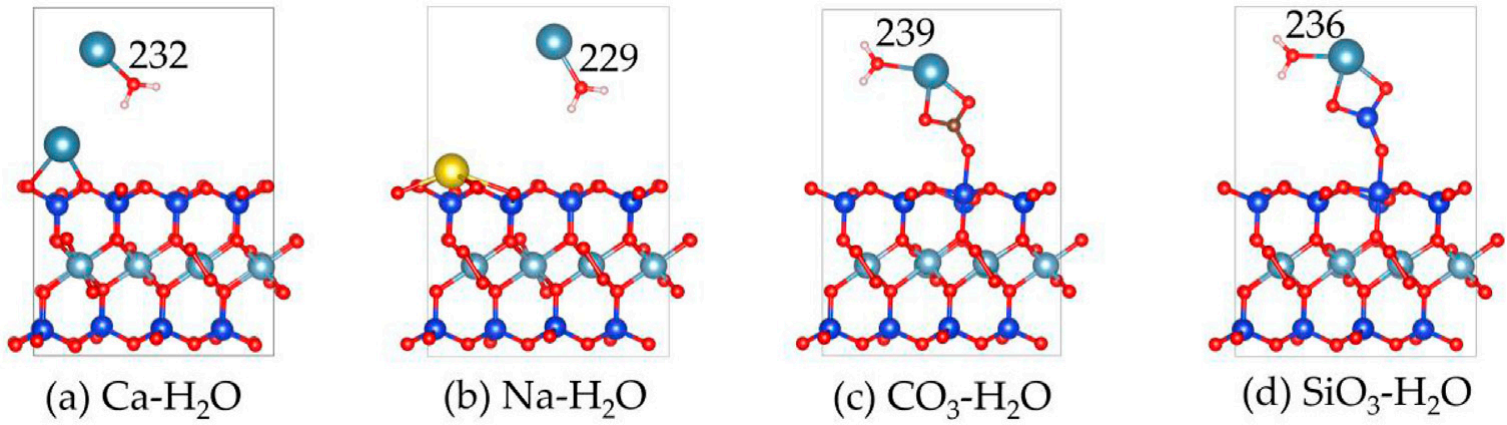
The structures of H_2_O on the **(a)** Ca^2+^, **(b)** Na^+^, **(c)** CO_3_^2-^ and **(d)** SiO_3_^2-^ absorbed montmorillonite.

**FIGURE 10:**
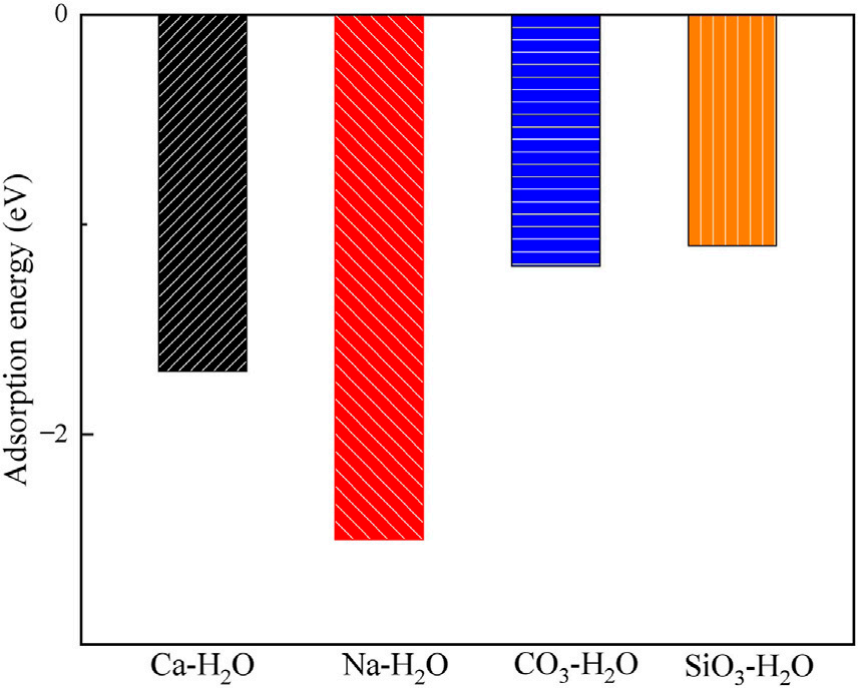
The adsorption energies of H_2_O on the Ca^2+^, Na^+^, CO_3_^2-^ and SiO_3_^2-^ absorbed montmorillonite.

Figures 9, 10 display the structures and adsorption energies for the adsorption of H_2_O on Ca^2+^, Na^+^, CO_3_^2-^, and SiO_3_^2-^-absorbed montmorillonite. The adsorption energy of Ca-H_2_O is −1.7 eV, which is lower than that of H_2_O on pristine montmorillonite (−3.1 eV). In this structure, the H_2_O molecule bonds with the layer Ca^2+^, with the distance between the O atom of H_2_O and the Ca^2+^ atom being 232 pm. For Na-H_2_O, the adsorption energy is −2.5 eV, which is stronger than Ca-H_2_O but comparable to H_2_O on clean montmorillonite. The H_2_O molecule bonds with surface Na^+^, with the O-Na bond distance being 229 pm.

In the CO_3_-H_2_O and SiO_3_-H_2_O structures, their adsorption energies are −1.2 eV and −1.1 eV, respectively. The adsorbed H_2_O molecules form bonds with Ca^2+^ through O, with Ca-O bond distances of 239 pm and 236 pm, respectively.

By comparing the adsorption of H_2_O on pristine montmorillonite and montmorillonite adsorbed with Ca^2+^, Na^+^, CO_3_^2-^, and SiO_3_^2-^, it is evident that montmorillonite adsorbed with Ca^2+^, Na^+^, CO_3_^2-^, and SiO_3_^2-^ inhibits water adsorption to different extents.

### 3.4 Adsorption mechanism from electronic structure

**FIGURE 11:**
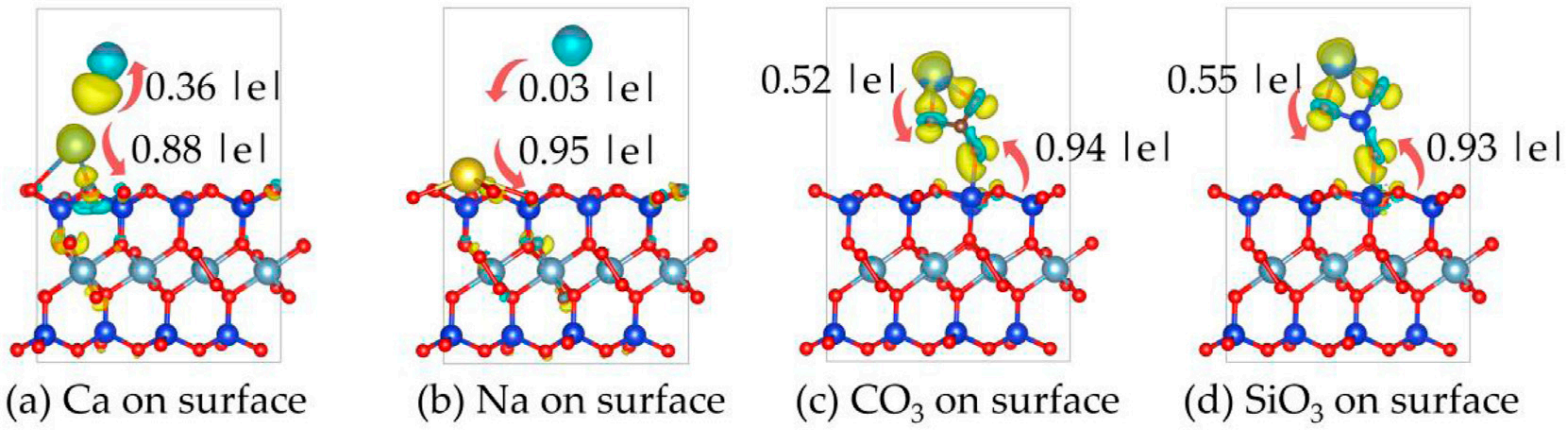
The differential charge density plots of **(a)** Ca^2+^, **(b)** Na^+^, **(c)** CO_3_^2−^ and **(d)** SiO_3_^2−^ on the montmorillonite surface. Yellow: charge accumulation; Cyan: charge depletion. The isosurface value is set to 0.008 e/Bohr3.

Figure 11 shows the differential charge density plots of Ca^2+^, Na^+^, CO_3_^2-^ and SiO_3_^2-^ on the on the montmorillonite surface.

It can be observed that there are significant changes in charge densities near the Ca^2+^ atoms and the surface O atoms of montmorillonite. Ca^2+^ atoms tend to lose electrons, characterized by a decrease in electron-cloud density (blue regions) around them. In contrast, the electron - cloud density around the surface O atoms that bond with Ca^2+^ increases (yellow regions). This indicates that during the adsorption of Ca^2+^, electrons are transferred from Ca^2+^ atoms to the surface atoms, forming ionic or polar covalent bonds. Such charge transfer enhances the interaction between Ca^2+^ and the montmorillonite surface. During the adsorption of Na^+^, the charge transfer is less intense compared to that of Ca^2+^. Therefore, in the figure, charge accumulation is only shown between the Na-O bonds.

When CO_3_^2-^ is adsorbed on the surface, there are notable changes in charge densities in the bonding regions between CO_3_^2-^ and the Ca^2+^ and Si atoms on the montmorillonite surface. There is an overlap and redistribution of electron clouds between the O atoms in CO_3_^2-^ and the Ca^2+^ and Si atoms. The increase in electron-cloud density around the O atoms indicates the inflow of electrons, leading to the formation of chemical bonds.

When SiO_3_^2-^ is adsorbed on the surface, significant changes in electron-cloud densities occur at the bonding sites between the O atoms of SiO_3_^2-^ and the Ca^2+^ and Si atoms on the montmorillonite surface. The electron-cloud enrichment around the O atoms (yellow regions) indicates that electrons are transferred from the surface atoms to the O atoms, forming stable chemical bonds.

Table 3 shows the charge transfer between Ca^2+^, Na^+^, CO_3_^2-^ and SiO_3_^2-^ with the montmorillonite surface. Ca^2+^ and Na^+^ exhibit significantly stronger charge transfer with surface atoms than with interlayer Ca. Notably, the charge transfer between Na^+^ and interlayer Ca^2+^ is extremely weak, with a magnitude of only 0.03|e|. The charge transfer trends highlight that Ca^2+^ and Na^+^ primarily interact with montmorillonite surfaces through cationic charge donation, with Ca^2+^ exhibiting stronger binding due to higher charge transfer. In contrast, both CO_3_^2-^ and SiO_3_^2-^ demonstrate substantial charge transfer with both surface and interlayer Ca^2+^ atoms. CO_3_^2-^ and SiO_3_^2-^ form stable associations with both surface and interlayer Ca^2+^ via electron acceptance, consistent with the structural insights from differential charge density plots.

**TABLE 3:** Charge transfer between Ca^2+^, Na^+^, CO_3_^2-^ and SiO_3_^2-^ with the montmorillonite surface.

	Ca^2+^	Na^+^	CO_3_^2-^	SiO_3_^2-^
Charge	1.24	0.92	−1.46	−1.48

### 3.5 Comparison with alkaline OH treatment

**FIGURE 12:**
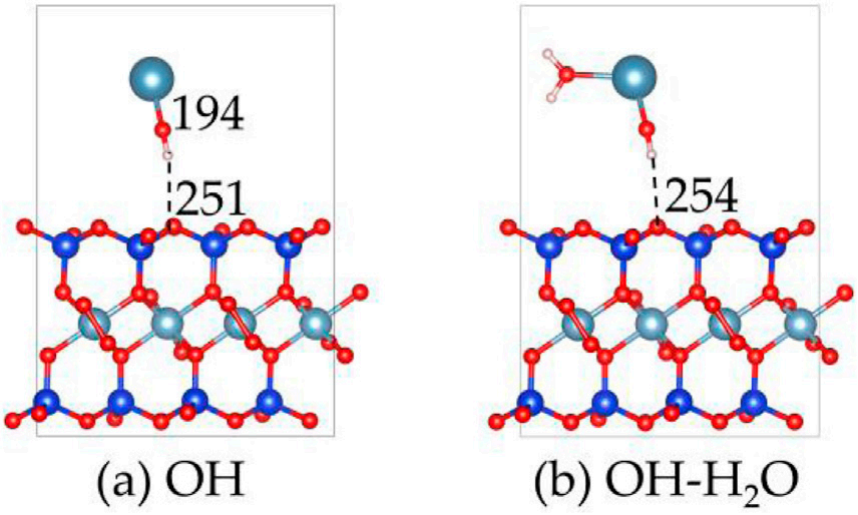
The structures of **(a)** OH on the montmorillonite, and the structures of **(b)** H_2_O on the OH absorbed montmorillonite.

**FIGURE 13:**
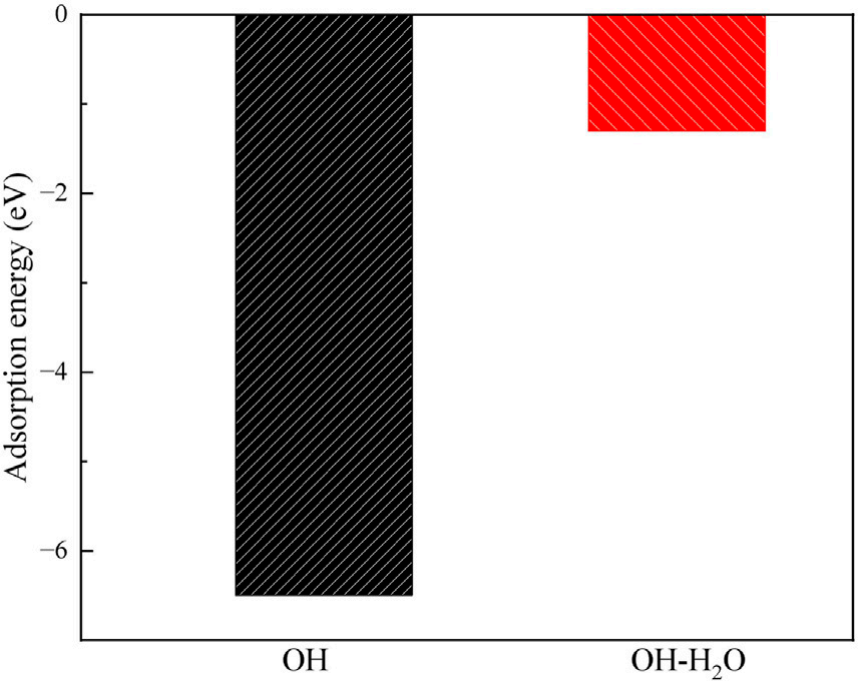
The adsorption energy of OH on the montmorillonite, and the adsorption energy of H_2_O on the OH absorbed montmorillonite.

The alkaline treatment process of montmorillonite has also been examined as shown in Figures 12, 13. The OH group adsorbs onto the Ca^2+^ atom in the layer, forming a Ca-O bond with a bond length of 194 pm. Additionally, the OH group forms a hydrogen bond with the surface, with a bond length of 251 pm, indicating the formation of a moderately strong hydrogen bond. The adsorption energy of the OH group is −6.5 eV, which is close to the −6.8 eV of CO_3_^2-^, but weaker than the −8.7 eV of SiO_3_^2-^.

Upon comparing the adsorption behaviors of hydroxide ions, carbonate ions, and silicate ions on the montmorillonite surface, it is observed that hydroxide ions primarily adsorb by forming hydrogen bonds with the surface, while carbonate and silicate ions adsorb via oxygen-silicon bonds. Since the bond energy and stability of hydrogen bonds are weaker than those of oxygen-silicon bonds, this is one of the reasons why the strength of lime-treated soils is often lower than that of soils stabilized with lime combined with sodium silicate or sodium carbonate.

Furthermore, the hydrophilicity of OH-absorbed montmorillonite was calculated, revealing that its H_2_O adsorption energy is −1.3 eV. This value is weaker than the −2.6 eV of molecular adsorption (Figure 7b) and the −3.1 eV of dissociative adsorption (Figure 7c), indicating that after the absorption of OH, the water affinity of montmorillonite decreases.

The formation of a moderately strong hydrogen bond and the reduction in water affinity help explain why lime treatment can enhance the strength and water stability of soils.

### 3.6 The effect of CO_2_ on stabilization

**FIGURE 14:**
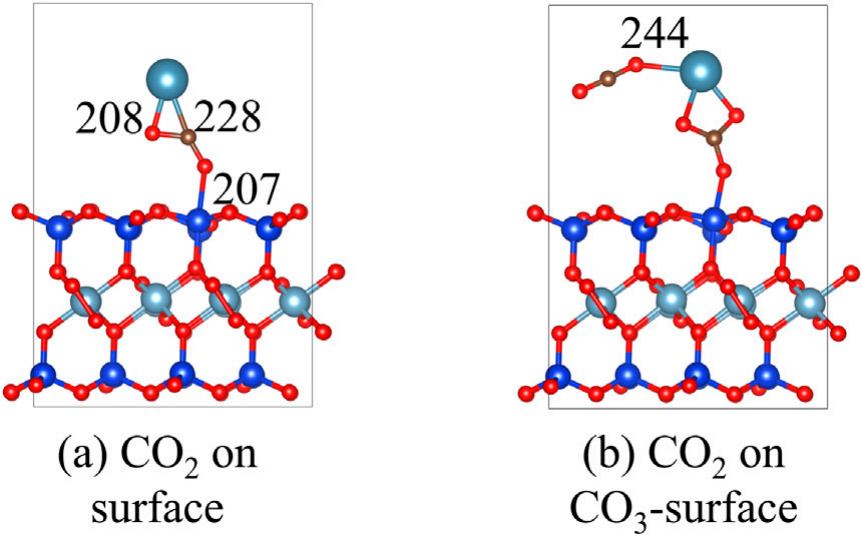
The structures of CO_2_ on the **(a)** Ca-montmorillonite and **(b)** CaCO_3_-montmorillonite surface.

The structures of CO_2_ on the Ca-montmorillonite and CaCO_3_-montmorillonite surface are shown in Figure 14. The adsorption energy of CO_2_ on the Ca-montmorillonite surface is calculated to be −0.2 eV. When CaCO_3_ forms on the montmorillonite surface, the adsorption energy of CO_2_ increases to −0.5 eV. These theoretical calculations suggest that the adsorption of CO_2_ by Ca-montmorillonite is thermodynamically favorable and is further enhanced by the nucleation of surface CaCO_3_. This is consistent with the findings of Song et al. (2024), who reported that increased carbonate content promotes CO_2_ storage.

In addition, Liu et al. (2025) reported that increased water content promotes CO_2_ adsorption. Under CO_2_-rich water conditions in deep underground environments, carbonate minerals can dissolve and recrystallize (Song et al., 2024), potentially affecting the stability of stabilized montmorillonite. This study focuses on improving the stability of stabilized montmorillonite near the Earth’s surface, where both pressure and temperature are relatively low. Although the adsorption energy for CO_2_ increases after nucleation, in practical engineering applications, the stabilizer-soil mixture is typically compacted at optimum water content, and the reaction products fill the pore spaces, increasing hydrophobicity. This significantly reduces water permeability of the stabilized soils, making it difficult to establish a CO_2_-rich water environment, thereby minimizing the risk of compromising stability and integrity.

### 3.7 Stabilization and hydrophilicity mechanisms

**FIGURE 15:**
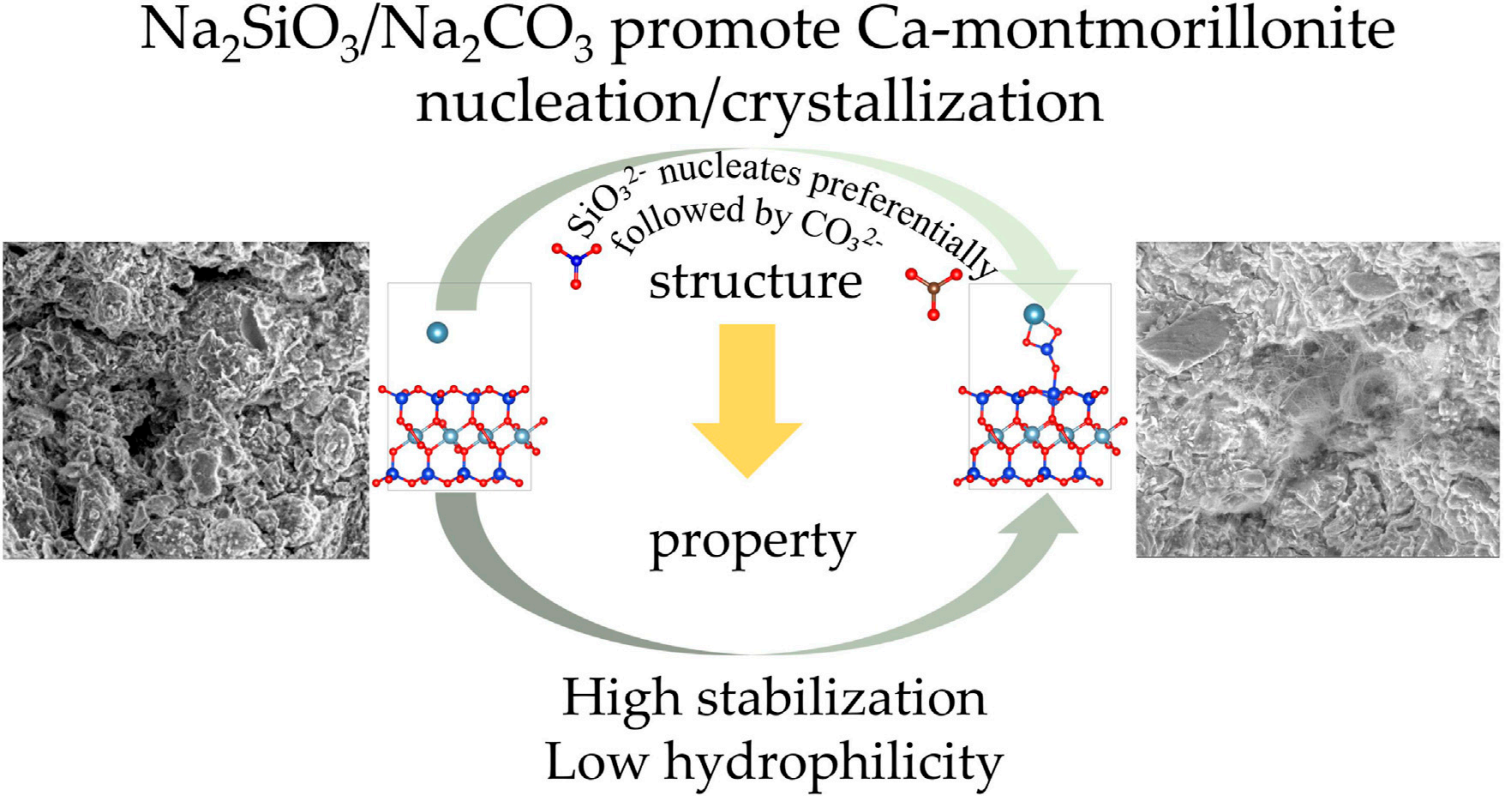
The nucleation, stabilization and hydrophilicity mechanisms schematic diagram.

The schematic diagram illustrating the mechanisms of nucleation, stabilization, and hydrophilicity is shown in Figure 15. When CO_3_^2-^ or SiO_3_^2-^ ions are present in the Ca-montmorillonite system, they preferentially nucleate and form precipitates on the surface of montmorillonite rather than adsorb onto the interlayer calcium ions. This is because, compared to adsorption on interlayer calcium ions (Figures 3c,d), the adsorption energy is lower when the calcium ions are adsorbed and nucleation occurs on the montmorillonite surface (Figures 5c,d). The lower the adsorption energy, the more stable the system becomes. Therefore, it can be speculated that, when free calcium ions, CO_3_^2-^ ions, and SiO_3_^2-^ ions coexist in the calcium-based stabilized soil system, these ions tend to form a spatial network structure of calcium carbonate and calcium silicate on the montmorillonite surface and within the pores through the sharing of calcium ions. This process enhances the overall integrity of montmorillonite, thereby improving its shear strength. This hypothesis aligns with the results observed in the SEM images (Figure 2).

Before stabilization, except for physical absorption (Figure 7a), the adsorption energies of water molecules in the interlayers of montmorillonite are relatively negative, and they are prone to adsorb through molecular absroption (Figure 7b) and dissociative adsorption (Figure 7c). This explains the strong hydrophilicity of montmorillonite, volume expansion after water absorption, and reduced macroscopic engineering properties. After stabilization, Na^+^ has almost minor effect on the hydrophilicity of montmorillonite, while Ca^2+^, CO_3_^2-^ or SiO_3_^2-^ significantly reduce its hydrophilicity. This can explain that calcium-based stabilizer alone, or in combination with Na_2_CO_3_ and Na_2_SiO_3_ can ruduce the affinity of montmorillonite for water.

By comparing the water adsorption energy of Ca-montmorillonite (Figure 5a) and Na-montmorillonite (Figure 5b), the calcium ions released by calcium-based stabilizer will exchange with Na^+^ and other ions through ion exchange, thus reducing the thickness of the water film. However, by comparing the reduction in water adsorption energy between montmorillonite adsorbed with calcium ions (Figure 9a) and montmorillonite adsorbed with calcium carbonate (Figure 9c) and calcium silicate (Figure 9d), it is evident that the improvement in water stability due to ion exchange is weaker than the improvement by the formation of precipitates.

The results above demonstrate that DFT calculations can effectively and quantitatively elucidate the interaction mechanisms between stabilizers and montmorillonite from the perspective of adsorption energy and electron transfer. This method can transform the selection of stabilizer components from a trial-and-error approach into a more targeted process based on the understanding of the interactions between clay minerals and candidates, reducing time-consuming, labor-intensive macro-mechanical tests and saving experimental costs. The DFT method is expected to not only enhance material screening for soil stabilization but also have broader applications in areas such as carbon sequestration, landfills, and beyond.

## 4 Conclusion

In this work, experiments and DFT calculation were used to investigate the stabilization and hydrophilicity mechanisms of Ca-montmorillonite systems modified with Na_2_CO_3_ and Na_2_SiO_3_.

1. SiO_3_^2-^ and CO_3_^2-^ ions preferentially adsorb on the montmorillonite surface rather than the exchangeable interlayer, with significantly higher adsorption energies compared to Ca^2+^ and Na^+^ ions.
2. The nucleation mechanism involves the bonding of O atoms from SiO_3_^2-^ and CO_3_^2-^ ions to the montmorillonite surface Si and Ca^2+^ ions in the interlayer, leading to the formation of CaSiO_3_ and CaCO_3_ network structures.
3. Adsorption of Ca^2+^, Na^+^, CO_3_^2-^, and SiO_3_^2-^ ions on the montmorillonite reduces the adsorption of H_2_O, lowering the water adsorption energy from −3.1 eV to −1.1 to −2.5 eV.
4. Na_2_CO_3_ and Na_2_SiO_3_ enhance the nucleation of CaSiO_3_ and CaCO_3_ on the montmorillonite surface, improving interfacial interactions and reducing water affinity. This has potential applications in reducing caprock permeability in saline aquifers for CO_2_ storage and improving the compressive strength and water resistance of stabilized soils in soil stabilization.

## Data Availability

The original contributions presented in the study are included in the article/supplementary material, further inquiries can be directed to the corresponding author.
